# Increase of Collectivistic Expression in China During the COVID-19 Outbreak: An Empirical Study on Online Social Networks

**DOI:** 10.3389/fpsyg.2021.632204

**Published:** 2021-04-20

**Authors:** Nuo Han, Xiaopeng Ren, Peijing Wu, Xiaoqian Liu, Tingshao Zhu

**Affiliations:** ^1^CAS Key Laboratory of Behavioral Science, Institute of Psychology, Chinese Academy of Sciences, Beijing, China; ^2^Department of Psychology, University of Chinese Academy of Sciences, Beijing, China

**Keywords:** collectivism, pathogen-prevalence hypothesis, online social networks, big data, COVID-19

## Abstract

The pathogen-prevalence hypothesis postulates that collectivism would be strengthened in the long term in tandem with recurrent attacks of infectious diseases. However, it is unclear whether a one-time pathogen epidemic would elevate collectivism. The outbreak of COVID-19 and the widespread prevalence of online social networks have provided researchers an opportunity to explore this issue. This study sampled and analyzed the posts of 126,165 active users on Weibo, a leading Chinese online social network. It used independent-sample *t*-tests to examine whether COVID-19 had an impact on Chinese collectivistic value-related behaviors by comparing the usage frequency of personal pronouns, group-related words, and relationship-related words before and after the outbreak. Overall, most collectivist words exhibited a significant upward trend after the outbreak. In turn, this tendency pointed to a rising sense of collectivism (versus individualism). Hence, this study confirmed the pathogen-prevalence hypothesis in real settings, finding that an outbreak of an infectious disease such as COVID-19 could exert an impact on collectivism and may deliver a theoretical basis for psychological protection against the threat of COVID-19. However, further evaluation is required to ascertain whether this trend is universal or culture-specific.

## Introduction

The outbreak of COVID-19 has resulted in an ongoing pandemic and has become a global public health crisis. Economies, societies, and politics across the world have felt the impact of COVID-19 for several months (Mehta et al., 2020). Social distancing, lockdowns, and other isolating actions have been suggested and adopted as protective behavioral mechanisms facilitating the avoidance of parasitic transmission along with other methods of managing local parasitic infections (Parmet and Sinha, 2020; Prem et al., 2020; Viner et al., 2020). Because the time period of this study selected to explore is prior to the time of WHO’s definition of COVID-19 as a pandemic, this study referred to coronavirus an epidemic instead of a pandemic. The hypothesis of pathogen prevalence (or the parasite stress model) hypothesizes that in the long term, inhabitants in regions with higher rates are more likely to become collectivist than the populations of regions that evince lower rates of infections (Fincher et al., 2008; Thornhill et al., 2010; Murray et al., 2011). However, could a one-time outbreak of an infectious disease such as COVID-19 also enhance behaviors displaying collectivist values? There is little direct evidence of such an assumption in real settings. An increasing number of people spent more time on online social networks after the outbreak of COVID-19 to obtain epidemic information on the epidemic and to express their concerns. This inclination provided the authors the opportunity to track the changes in behaviors displaying collectivist values (e.g., collectivistic expression) with time cues following the outbreak of COVID-19 (Liu M. et al., 2018; Holmes et al., 2020; Li S. et al., 2020). The researchers engaged in this study were also inspired to examine whether the outbreak of COVID-19 triggered concerns toward ingroup members, a trend that could imply the growth of collectivist values. Results congruent with the abovementioned postulation would offer new evidence supporting the pathogen-prevalence hypothesis. Further, such a study may deliver a theoretical basis for psychological protection against the threat of COVID-19 (Germani et al., 2020).

Individualism/collectivism, as a fundamental cultural dimension, captures cultural perspectives in people’s differentiation toward ingroups and outgroups (Oyserman et al., 2002; Fincher and Thornhill, 2012; Oyserman, 2017; Van de Vliert, 2020). The pathogen-prevalence hypothesis is thought to cause geographical or cultural differences in individualism/collectivism (Fincher et al., 2008). The pathogen-prevalence hypothesis postulates that in the long term, collectivistic values, such as ingroup–outgroup differentiation, ingroup favoritism, and outgroup xenophobia, would be strengthened in tandem with recurrent attacks of infectious diseases (Oyserman et al., 2002; Fincher and Thornhill, 2008a; Fincher and Thornhill, 2008b; Fincher and Thornhill, 2012; Fincher et al., 2008; Oyserman, 2017). This hypothesis is supported by substantial cross-cultural evidence. For example, Fincher and his colleagues drew on epidemiological data and the findings of worldwide cross-national surveys of individualism/collectivism finally found that the regional prevalence of pathogens evinces an extremely positive correlation with cultural indicators of collectivism and exhibits a strong negative correlation with individualism (Fincher et al., 2008). The severity of parasitical illnesses could also positively predict collectivist-value-related behavior, for example, family ties, xenophobia, philopatry (Fincher and Thornhill, 2012), obedience (Cashdan and Steele, 2013), and ingroup trust (Zhang, 2018). However, some of the evidence proffered by these studies did not exclude the interference caused by the confounding factors emanating from cross-cultural studies, such as interferences caused by varying degrees of modernization, and diverse social systems. Thus, it is difficult to establish a causal link between the severity of the localized outbreak of a parasite disease and the growth of collectivist sentiments (Yang, 1988; Oyserman et al., 2002; Kagitcibasi, 2005). Some scholars manipulated the exposure to pathogen cues and found that exposure to pathogen cues could elevate ethnocentrism (Navarrete et al., 2007), conformity (Wu and Chang, 2012), and outgroup prejudice (Tybur and Lieberman, 2016). Nevertheless, these extant experimental investigations could not guarantee the ecological validity like cross-cultural studies. Moreover, scholars also found that during a parasitic disease outbreak, collectivist-value-related behaviors buffered adverse outcomes in instances of outbreaks of parasitic disease. For example, Kim tested the influence of collectivism on xenophobic response to the threat of Ebola and found that collectivism—and the set of practices and rituals associated with collectivistic cultures—may serve as psychological protection against the threat of disease (Kim et al., 2016). All these findings lend impetus and support to the present study to employ an ecological method to explore whether a one-time outbreak of an infectious disease such as COVID-19 could enhance behaviors that showcase collectivist values in real settings and therefore to provide a theoretical basis for psychological protection against the threat.

The COVID-19 pandemic and the widespread prevalence and use of online social networks (OSNs) represented an opportunity for the present study. Various news outlets continuously warned their users officially and unofficially after the outbreak of COVID-19, labeling it as an extremely serious infectious disease. Many countries enforced and suggested interventions, such as isolation (Hellewell et al., 2020), quarantining (Parmet and Sinha, 2020), school closures (Viner et al., 2020), social distancing (World Health Organization, 2020a), and mask wearing (Feng et al., 2020). Individuals were also warned to perceive and experience the secondary threat of COVID-19. All these interventions and sources of information prompted people to use OSNs to demonstrate their concerns and express their feelings (Cinelli et al., 2020; Gao et al., 2020; Li S. et al., 2020).

The popularity and the proliferation of OSNs have encouraged extensive social interactions among users and have generated a large amount of social data. OSNs have been used to explore personal, societal, and cultural outcomes and represent rich resources for the apprehension of underlying psychological mechanisms. There were 3.08 billion registered social media network users worldwide as of 2020 (Statista, 2020a. Evidence has shown that people’s digital records on social media are extended into real life and might be an efficient medium for expressing and communicating real personality traits (Back et al., 2010). Kosinski et al. (2013) utilized a dataset of over 58,000 volunteers who offered access to their Facebook likes and detailed demographic profiles and examined the results of several psychometric tests. Their study determined that Facebook likes could be used to automatically and accurately predict a range of highly sensitive personal attributes, including sexual orientation, ethnicity, religious and political views, personality traits, intelligence, happiness, use of addictive substances, parental separation, age, and gender (Kosinski et al., 2013). Other researchers have found that people living in individualist cultures were less egocentric in social networks than those residing in collectivist societies (Na et al., 2015). Moreover, individuals belonging to culturally tight (versus loose) states were more likely to express positive emotions and were less likely to express negative emotions (Liu P. et al., 2018). There are also some previous studies that have investigated the collectivism and social media postings (, Arpaci and Baloğlu, 2016; Arpaci et al., 2018, 2020). The investigation selected the leading Chinese OSN, Sina Weibo, which has 516 million registered users (Statista, 2020b), as its analytics platform. All of the microblogs on Sina Weibo are publicly available and can be utilized to recognize individual psychological traits and to ascertain mental health statuses (Hao et al., 2015; Li S. et al., 2020), analyze emotional states (Liu M. et al., 2019), and apply the suicide ideation test (Li A. et al., 2018).

It is difficult to conduct a traditional survey or perform wide-ranging experimental manipulations during the ongoing COVID-19 epidemic. Also, it was impossible to measure collectivism in people in advance since the timing of the COVID-19 declaration was unknown. Moreover, the digital records of human behavior from OSNs can offer more ecological validity than classic psychological surveys and experimental manipulations. Thus, problems of documentation may be avoided, and real-time, non-invasive detection is made possible, ensuring the objectivity, timeliness, and continuity of the data.

In general, the outbreak of COVID-19 and the widespread use of OSNs accorded the researchers the opportunity to explore whether a one-time outbreak of an infectious disease would also advance behaviors displaying collectivist values in real settings. Meanwhile, the testing of the pathogen-prevalence hypothesis may present references for policymakers and help them plan and fight effectively against the COVID-19 pandemic. This study posits that the exposure to pathogen cues of COVID-19 has increased the usage of collectivist words and that people use such terms more after they know COVID-19 is infectious than before.

## Materials and Methods

### Participants and Data Collection

The present investigation was based on microblog text analyses. The active users were sampled from the original Weibo data pool (Li et al., 2014), which contained more than 1.16 million active users. The retrieved data included information on user profiles and posts. The privacy of users was strictly protected during this process according to the ethical principles reference listed by Kosinski et al. (2015). The ethics code is H15009 approved by the Institutional Review Board at the Institute of Psychology, Chinese Academy of Sciences.

The active users were defined as Weibo members (1) who published 10 or more original microblogs during the epidemic period, (2) whose authentication type was non-institutional (such as individual users, etc.), and (3) whose regional authentication was not blank. Moreover, users who had registered from overseas locations such as Hong Kong, Macao, or Taiwan were excluded from the study. Ultimately, 126,165 active users (94,436 men, 31,729 women; median age = 29) were selected from the 1.16 million Weibo users. The participants were spread across 481 cities in 31 provincial administrative regions in mainland China. Their original posts published between December 1, 2019, and February 16, 2020, were then fetched for analysis; each user posted an average of 109.5 microblogs.

### Word Selection

Language indicators were developed to measure individualism/collectivism, including pronouns (Kashima and Kashima, 1998; Twenge et al., 2013) and individualist/collectivist terms (Zeng and Greenfield, 2015). Pronouns have been proven to be indicators of individualism/collectivism. Kashima found that the pronouns employed in spoken language were positively predicted by collectivism among 71 cultures with 39 languages (Kashima and Kashima, 1998). First-person singular pronouns (I or me) have been linked to individualism; first-person plural pronouns (we or us) to collectivism in the “pronoun circle” paradigm (Oyserman and Lee, 2008) of cultural changes noted in Google Ngram Corpus database or other texts (Yu et al., 2016). Further, the second- and third-person pronouns have also been evidenced as potential indicators of individualism/collectivism. For example, Hamamura and colleagues found in Chinese that second- and third-person pronouns evinced similar trends in the Google Ngram Corpus database (Hamamura and Xu, 2015). It is suggested that singular pronouns are linked to individualism, and plural pronouns are associated with to collectivism (first-, second-, and third-person).

Two other kinds of words were analyzed to supplement the result and to explore whether the effects of COVID-19 on behaviors that presented collectivist values depended on the intimacy of ingroups. One is group-related words. In the collectivist Chinese culture, individuals are embedded in various groups, such as work units, communities, families, governments (Lu, 2012; Xie, 2016), and social networks (e.g., family, kinship, neighbor, acquaintances, colleagues). Interpersonal relationships are important to Chinese individuals, and all associations are not equal (Gold et al., 2002). Additionally, among all their groups, the Chinese cares most about their nuclear family, then their extended family, and then other kinship relations (Hwang, 1987). Good relationships with five cardinal groups (in Chinese, “五伦”) have been used in China as measures of relational harmony for the appraisal of specific cultural influence on life satisfaction (Kwan et al., 1997). This phenomenon implies that group-related words may denote a novel method of verifying whether collectivist expressions are increasing in China. The other is relationship-related words. Compared with other languages, there are more words to point to specific relationships in Chinese in comparison to other languages. For example, in Chinese, “father’s brother” could be “叔叔” or “伯伯,” and “mother’s brother” is “舅舅.” However, in English, all these relationships are represented by the term “uncle.” The specificity of relationship-related words may symbolize cultural differences that indicate the rigidity or laxity of the social structure and could be employed to verify whether collectivist expressions are increasing.

Finally, the frequencies of specific words were computed from the original posts published by the active users of Weibo. The words were selected based on the following methodology. (1) First-person singular pronouns, second-person singular pronouns, third-person singular pronouns, first-person plural pronouns, second-person plural pronouns, and third-person plural pronouns were selected on the basis of previously conducted research as the objects of analysis (Hamamura and Xu, 2015; Yu et al., 2016). (2) Word categories that can summarize varied groups, including family, kinship, neighbors, acquaintances, and colleagues (Oyserman et al., 2002), were selected by referencing previous methods of incorporating interpersonal relationships into individualism–collectivism research. (3) Further, the present investigation also selected relationship-related words including father, mother, son, daughter, sister, brother, uncle, aunt, niece, nephew, grandmother, grandfather, grandson, and granddaughter to explore the extent of attention people paid to family members during the epidemic (Murdock, 1949; Lu, 2012; Xie and Hu, 2014). The detailed classification of personal pronouns, group-related words, and relationship-related words are shown in, respectively, displayed in Table 1.

**TABLE 1:** The detailed information of collectivist words.

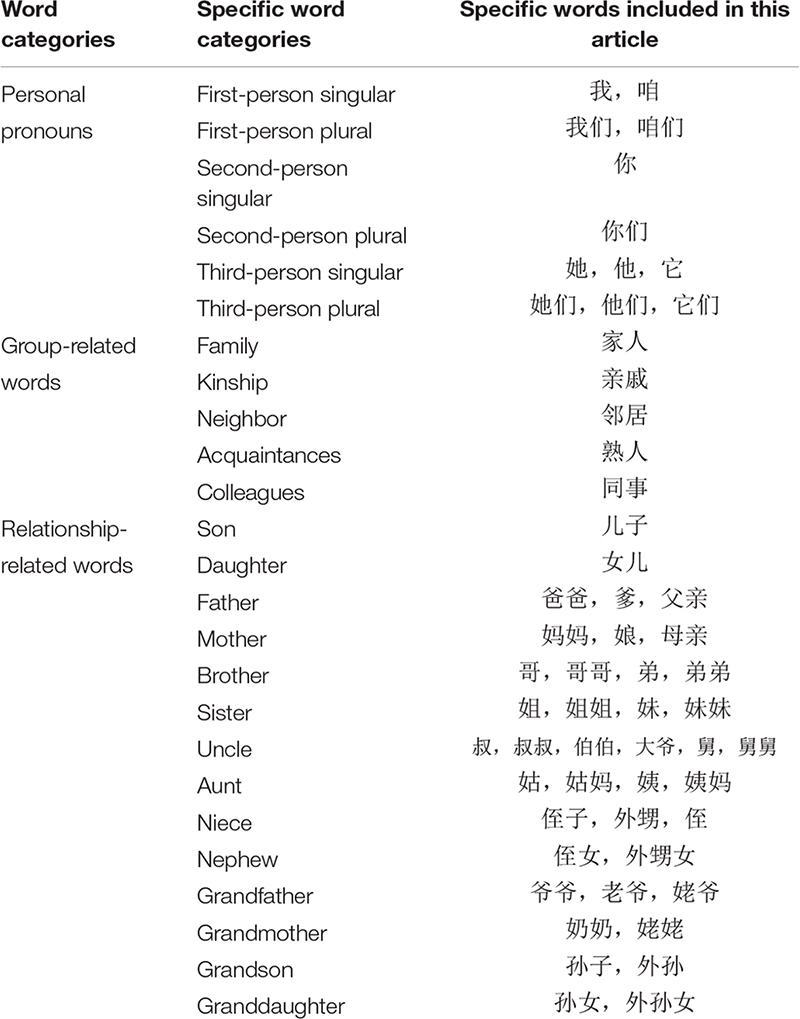

### Measures and Analysis

Original posts published by active Weibo users from December 1, 2019, to February 16, 2020, were fetched for analysis. This period was selected because China’s first case of COVID-19 appeared on December 1, 2019 (Huang et al., 2020), and the National Health Commission of China officially identified COVID-19 as a class B infectious disease—a disease which may cause epidemics and is considered mandatory a notifiable disease (Li Y. et al., 2020), on January 20, 2020 (National Health Commission of the People’s Republic of China, 2020). Some provinces in China began to sequentially resume work and production from February 10, 2020. The Joint Prevention and Control Mechanism of the State Council of China announced on February 16, 2020, that the proportion of severe cases diagnosed in the country had dropped significantly (World Health Organization, 2020b), which represented a positive sign that the situation had started to improve. Therefore, the selected time period was divided into two stages: Stage I (December 1, 2019–January 20, 2020) denoted the unclear stage of the epidemic when people were not aware of the infectiousness of COVID-19; Stage II (January 21, 2020–February 16, 2020) encompassed the severe stage of the epidemic. The Weibo data then divided into two parts from December 1, 2019, to January 20, 2020, and from January 21, 2020, to February 16, 2020 (hereinafter referenced as early stage and later stage).

**FIGURE 1:**
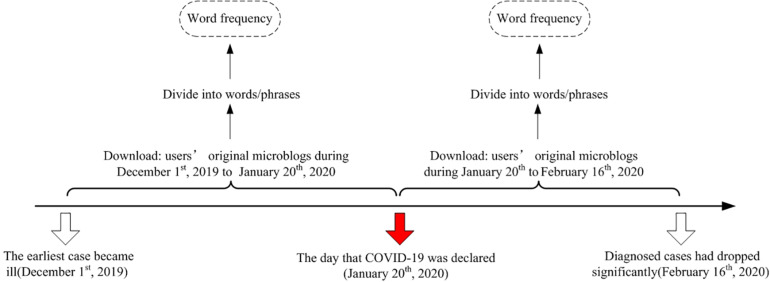
Procedure from feature extraction to word frequency.

The TextMind system developed by the Computational Cyber-Psychology Lab of the Institute of Psychology at the Chinese Academy of Sciences was used to extract the text features for this study (Gao et al., 2013). The Chinese word segmentation embedded in the TextMind system can divide the text into independent words using linguistic characteristics in accordance with the rules of Chinese grammar rules, to finally achieve the purpose of analyzing word frequencies using computers. The counts of each term were obtained on the basis of a psychoanalysis dictionary, and the ratio of the number of occurrences of each word was then computed vis-à-vis the total number of words used each day to control the impact of daily total word counts changes. Figure 1 portrays the procedures adopted from feature extraction to word frequency. Therefore, the analysis of the change trend of each word during the epidemic was more accurately accomplished. Finally, we compared the differences between all word frequencies in early and later stages through independent-sample *t*-tests by using the Statistical Product and Service Solutions (SPSS) 22.0 (Corp, 2013) for data analysis.

## Results

### Personal Pronouns

In this study, we compared the word frequency of personal pronouns between early and later stages. The detailed information of personal pronouns is seen in Table 1. The independent-sample *T*-test results for personal pronouns are shown in Table 2. After the outbreak of COVID-19, word frequency significantly increases in the category plural pronouns, including first-person plural pronouns (*t* = −9.12, *p* < 0.001, *d* = 2.35), second-person plural pronouns (*t* = −9.41, *p* < 0.001, *d* = 0.72), and third-person plural pronouns (*t* = −9.20, *p* < 0.001, *d* = 0.67). Word frequency significantly decreases in the category singular pronouns, including first-person singular pronouns (*t* = 5.62, *p* < 0.001, *d* = 1.26) and second-person singular pronouns (*t* = 8.18, *p* < 0.001, *d* = 3.16).

**TABLE 2:** Comparison of personal pronouns between early and later stages.

Pronouns categories	Early stage (*N* = 50)		Later stage (*N* = 28)		*T*	*p*	*Cohen’s d*
	*M*	*SD*	*M*	*SD*			
First-person singular	4502.60	366.35	3926.43	535.64	5.62	0.000***	1.26
First-person plural	242.05	30.94	368.15	69.41	–9.12	0.000***	2.35
Second-person singular	1454.14	171.12	1071.12	141.67	8.18	0.000***	3.16
Second-person plural	96.27	14.64	156.21	31.88	–9.41	0.000***	0.72
Third-person singular	532.59	52.79	523.76	86.55	0.56	0.577	0.12
Third-person plural	69.46	10.35	121.44	28.87	–9.20	0.000***	0.67

“Early stage” represents the word frequency from December 1, 2019, to January 20, 2020. “Later stage” represents the word frequency from January 21, 2020 to February 16, 2020. ***p < 0.001.

### Group-Related Words

We also found significant differences in group-related words (see detailed information in Table 1) between early and later stages. As shown in Table 3, after the outbreak of COVID-19, a small part of word frequency significantly decreased in the group-related words, including colleagues (*t* = 3.80, *p* < 0.001, *d* = 0.95) and acquaintances (*t* = 2.45, *p* = 0.018, *d* = 0.49). Most of word frequency significantly increased in the category group-related words, including family (*t* = −4.39, *p* < 0.001, *d* = 1.10), kinship (*t* = −2.85, *p* = 0.008, *d* = 0.75), and neighbor (*t* = −2.85, *p* = 0.008, *d* = 1.04).

**TABLE 3:** Comparison of group-related words between early and later stages.

Categories	Early stage (*N* = 50)		Later stage (*N* = 28)		*T*	*p*	*Cohen’s d*
	*M*	*SD*	*M*	*SD*			
Family	23.79	11.68	41.84	19.94	–4.39	0.000***	1.10
Kinship	2.82	1.94	6.61	6.90	–2.85	0.008**	0.75
Neighbor	2.75	1.05	5.77	3.99	–3.94	0.000***	1.04
Acquaintances	1.82	3.14	0.70	0.65	2.45	0.018*	0.49
Colleagues	17.43	6.88	11.94	4.41	3.80	0.000***	0.95

“Early stage” represents the word frequency from December 1, 2019, to January 20, 2020. “Later stage” represents the word frequency from January 21, 2020, to February 16, 2020. ***p < 0.001, **p < 0.01, *p < 0.05.

### Relationship-Related Words

Results indicate significant differences of relationship-related words (see detailed information in Table 1) between early and later stages, as shown in Table 4. The word frequency of mother (*t* = −1.93, *p* = 0.058, *d* = 0.44) and niece (*t* = −1.89, *p* = 0.063, *d* = 0.42) significantly increased in marginal, while the word frequency of uncle (*t* = −2.67, *p* = 0.009, *d* = 0.59), grandfather (*t* = −2.40, *p* = 0.022, *d* = 0.61), and grandmother (*t* = −3.29, *p* = 0.002, *d* = 0.76) significantly increased. However, the word frequency of son (*t* = 2.86, *p* = 0.006, *d* = 0.65), brother (*t* = 3.82, *p* < 0.001, *d* = 0.90), and sister (*t* = 6.87, *p* < 0.001, *d* = 1.68) significantly decreased.

**TABLE 4:** Comparison of relationship-related words between early and later stages.

Categories	Early stage (*N* = 50)		Later stage (*N* = 28)		*T*	*p*	*Cohen’s d*
	*M*	*SD*	*M*	*SD*			
Son	23.12	5.24	19.24	6.59	2.86	0.006**	0.65
Daughter	15.38	4.24	14.38	12.29	0.42	0.678	0.11
Father	55.95	12.34	55.04	12.67	0.309	0.758	0.07
Mother	118.66	15.97	126.74	20.60	–1.93	0.058	0.44
Brother	221.49	30.23	193.64	31.98	3.82	0.000***	0.90
Sister	167.33	25.40	129.31	19.36	6.87	0.000***	1.68
Uncle	36.06	9.72	43.74	15.68	–2.67	0.009**	0.59
Aunt	43.27	10.72	41.84	6.06	0.75	0.456	0.16
Niece	1.23	1.09	1.76	1.40	–1.89	0.063	0.42
Nephew	1.80	1.32	1.75	1.17	0.17	0.867	0.04
Grandfather	16.38	7.57	23.19	13.92	–2.40	0.022*	0.61
Grandmother	16.47	5.45	20.93	6.25	–3.29	0.002**	0.76
Grandson	1.71	0.99	1.82	1.65	–0.37	0.738	0.08
Granddaughter	0.57	0.33	2.47	9.46	–1.06	0.298	0.28

“Early stage” represents the word frequency from December 1, 2019, to January 20, 2020. “Later stage” represents the word frequency from January 21, 2020, to February 16, 2020. ***p < 0.001, **p < 0.01, *p < 0.05.

## Discussion

The present study used large-scale time-series data obtained from Sina Weibo to scrutinize the effects of COVID-19 on individual behaviors exhibiting collectivist values. As predicted, individuals were more inclined to use words related to collectivist values during the later stage and employed lesser terms displaying individualist values than the early stage. Specifically, individuals preferred to use more plural pronouns as well as group-related and relationship-related words and less singular pronouns during the later stage. These results lend support to the pathogen-prevalence hypothesis of collectivism, which posits that inhabitants tend to embrace collectivist values to protect themselves behaviorally from threats. In the course of the epidemic, individuals cared more about ingroup members and relied more on them to defend against the serious threat posed by the infectious diseases. In addition, behaviors related to collectivist values relied on the closeness of ingroups during the impact of COVID-19. At this juncture, colleagues and acquaintances were relatively less important than family members or significant ingroup members.

This study reports opposing results that could be attributed to the limitations of its data and/or factors not considered in its assumptions. First, the decrease of mentions of acquaintances and colleagues may be caused by the fact that the family represents the most important group, followed by kinship networks, neighbors, work colleagues, and acquaintances. These units form types of an individual’s ingroup, but there exists an ingroup vigilance or peer pressure between work colleagues and acquaintances (Liu S. S. et al., 2019). Second, some relationship-related words indexing collectivistic values declined in frequency; these included son, brother, and sister. This outcome may be explained by the generalization of the kinship terms (Ren and Chen, 2019). The features of authority and gravity embedded in the numerous kinship morphemes in traditional cultures have gradually faded because of the influence of the openness of social culture and the diversification of online languages. The current generation commands a high degree of openness and entertainment. Many kinship terms can now be used as entertaining self-appellations (Cha and Gao, 2011; Wang, 2011; Xie, 2018) or be utilized by groupies (e.g., call idol “哥哥” or “姐姐”). The number of entertainment-related microblogs decreased in the later stage, perhaps symbolizing that Chinese individuals were more concerned about information on the epidemic.

In sum, the results of this study contribute to the extant literature in four ways. First, the present investigation expands the pathogen-prevalence hypothesis. Most evidence of the parasite-stress theory has thus far emanated from cross-cultural correlations (Fincher et al., 2008; Schaller and Murray, 2008; Thornhill et al., 2010; Li S. et al., 2020; Thornhill and Fincher, 2011; Zhang, 2018). Previously conducted research has indicated that inhabitants acquired adapted behavioral immune systems through prolonged recurrences of infectious diseases (Thompson, 2005). Whether the behavior of a population would change temporarily during the outbreak of a parasitic disease was not clear. This investigation tested the hypothesis in a real setting. Moreover, the cross-cultural tests based on historical data may incorporate an inherent weakness because historical sources may be coarse-grained and less accurate than modern disease prevalence data (Cashdan and Steele, 2013). The current study’s results elucidate that the inhabitants of mainland China evinced observable collectivist behavioral transformations after the outbreak of an infectious disease.

Second, the study also contributes to cultural psychology through the use of big data, which can enhance our understanding of cultural psychology. Digital records of the behaviors of Sina Weibo constitute large-scale big data without the limitations of self-reports. These data evinced the link between collectivist behavior and the outbreak of COVID-19, and the ecological validity of this outcome is persuasive. Moreover, the Weibo results obtained by this study were adequately controlled for confounding factors such as the time window in comparison to the use of the Google Ngram Corpus for the analyses of collectivism (Zeng and Greenfield, 2015; Yu et al., 2016).

Third, the present investigation attempted to expand collectivist terminology to supplement the results. Pronouns, especially first-person, were used as indicators of individualism/collectivism (Kashima and Kashima, 1998; Hamamura and Xu, 2015). This idea was developed from cross-cultural comparisons in which some culture- or language-specific words were neglected. In this instance, two types of collectivist-related words were defined and constructed in congruence with the collectivist definitions and characteristics of the Chinese language: group-related and relationship-related words. The two kinds of collectivist terms evinced the same trends in this study along with the personal plural pronoun, offering preliminary evidence that these two types of words may be utilized as indicators of collectivism. More rigorous tests of these two types of terms could be performed in future investigations.

Finally, the current study tested the pathogen-prevalence hypothesis in the circumstances of a real epidemic. Such a basis may deliver a theoretical basis for psychological protection against the threat of COVID-19 (Germani et al., 2020) and may help policymakers to plan and fight against COVID-19 more effectively (). Although previous studies testing the pathogen-prevalence hypotheses command more internal validity, the current investigation was able to expand the external validity.

Some limitations of this study must, however, be acknowledged. First, this investigation pertains only to the trend toward collectivism for the duration of an outbreak. It remains to be explored whether the frequencies of such collectivist words will fall to the same normal levels after the epidemic as before the outbreak of COVID-19. Second, trends toward behaviors displaying collectivist values were observed by this study; however, it is uncertain whether this trend is universal or culture-specific. Third, this study only used a longitudinal design for Chinese culture using Weibo; the examination of its results should be tested in other cultures based on other social media such as Twitter and Facebook. Fourth, the segmentation of Chinese characters is a challenging problem. For example, the first-person singular pronouns are sometimes also used to indicate first-person plural pronouns as in expressions such as “我国” (our country). Finally, many factors were not controlled in the study. For example, other events may relate to the collectivist words used on Weibo. In the future, these uncertain factors should be controlled using experimental methods, which would make the study more robust. In short, the present study is still imperfect; however, it does indicate that the data obtained from Weibo was able to yield observations of certain changes in Chinese collectivism.

## Conclusion

This study analyzed the frequencies of personal pronouns, group-related words, and relationship-related words in the early and later stages of COVID-19 on the basis of data obtained from Sina Weibo, a leading social media platform in China. The results of the study evince that first-person plural pronouns increased in frequency as the pandemic worsened; meanwhile, the word frequency of first-person singular pronouns deceased. Besides, Chinese individuals referred significantly more to group-related words and relationship-related words during the later stage of COVID-19 than in the early stage. Even though it is still indeterminate whether this trend is universal or culture-specific, the outcomes of this study indicate that an outbreak of an infectious disease such as COVID-19 could influence collectivism. Through this result, the present study is able to confirm the pathogen-prevalence hypothesis in a real setting. Moreover, study proved the validity of using data from OSNs for analyses in social–psychological research purposing to describe human behavior, especially in the context of culture.

## Data Availability Statement

Due to protect the privacy of the participants, the original posts used for the analysis are not publicly available but are available from the corresponding author on reasonable request.

## Ethics Statement

The studies involving human participants were reviewed and approved by the Institutional Review Board at the Institute of Psychology, Chinese Academy of Sciences. Written informed consent from the participants’ legal guardian/next of kin was not required to participate in this study in accordance with the national legislation and the institutional requirements.

## Author Contributions

NH, XR, and TZ conceived and planned this article. NH and XR carried out the search and revision of the literature. TZ collected and provided the data. NH and PW analyzed the data. NH drafted the study. XL, XR, and TZ reviewed and edited the writing. All authors revised the article critically for important intellectual content, commented on and approved the final manuscript, were accountable for all aspects of the work, read, and agreed to the published version of the manuscript.

## Conflict of Interest

The authors declare that the research was conducted in the absence of any commercial or financial relationships that could be construed as a potential conflict of interest.
